# A novel technique of reverse-sequence endoscopic nipple-sparing mastectomy with direct-to-implant breast reconstruction: medium-term oncological safety outcomes and feasibility of 24-h discharge for breast cancer patients

**DOI:** 10.1097/JS9.0000000000001134

**Published:** 2024-02-09

**Authors:** Jiao Zhou, Yanyan Xie, Faqing Liang, Yu Feng, Huanzuo Yang, Mengxue Qiu, Qing Zhang, Kawun Chung, Hui Dai, Yang Liu, Peng Liang, Zhenggui Du

**Affiliations:** a Department of General Surgery; b Breast Center; c Day Surgery Center, West China Hospital, Sichuan University; d Department of General Surgery, The Fourth People’s Hospital of Sichuan Province, Chengdu; e Department of Thyroid and Breast Surgery, The First People’s Hospital of Ziyang, Sichuan University, Ziyang, China

**Keywords:** breast cancer, direct-to-implant breast reconstruction (DIBR), discharge within 24 hours, oncologic safety outcomes, reverse-sequence endoscopic nipple-sparing mastectomy (R-E-NSM)

## Abstract

**Background:**

Due to the short operation time and no need for special instruments, reverse-sequence endoscopic nipple-sparing mastectomy (R-E-NSM) with direct-to-implant breast reconstruction (DIBR) has been rapidly becoming popular in the last three years. However, there has yet to be an evaluation of its oncologic safety or the feasibility of discharging patients within 24 h.

**Materials and methods:**

In this single-centre retrospective cohort study, individuals diagnosed with stage 0–III breast cancer between May 2020 and April 2022 who underwent traditional open mastectomy or R-E-NSM with DIBR were included. Follow-up started on the date of surgery and ended in December 2023. Data, including demographics, tumour characteristics, medium-term oncological outcomes, and postoperative complications, were collected and analyzed. Propensity score matching (PSM) was performed to minimize selection bias.

**Results:**

This study included 1679 patients [median (IQR) age, 50 [44–57) years]. Of these, 344 patients underwent R-E-NSM with DIBR (RE-R group), and 1335 patients underwent traditional open mastectomy (TOM group). The median [IQR] follow-up time was 30 [24–36] months [29 (23–33) months in the RE-R group and 30([24–36) months in the TOM group]. Regarding before or after PSM, the *P* value of local recurrence-free survival (LRFS, 0.910 and 0.450), regional recurrence-free survival (RRFS, 0.780 and 0.620), distant metastasis-free survival (DMFS, 0.061 and 0.130), overall survival (OS, 0.260 and 0.620), disease-free survival (DFS, 0.120 and 0.330) were not significantly different between the RE-R group and the TOM group. The 3y-LRFS and 3y-DFS rates were 99.0% and 97.1% for the RE-R group and 99.5% and 95.3% for the TOM group, respectively. The rates of any complications and major complications were not significantly different between the RE-R patients who were discharged within 24 h and the RE-R patients who were not discharged within 24 h (*P*=0.290, *P*=0.665, respectively) or the TOM patients who were discharged within 24 h (*P* =0.133, *P*=0.136, respectively).

**Conclusions:**

R-E-NSM with DIBR is an innovative oncologic surgical procedure that not only improves cosmetic outcomes but also ensures reliable oncologic safety and fewer complications, enabling patients to be safely discharged within 24 h. A long-term prospective multicenter assessment will be supporting.

## Introduction

Highlights

- In this study, reverse-sequence endoscopic nipple-sparing mastectomy (R-E-NSM) with direct-to-implant breast reconstruction (DIBR) was compared with traditional mastectomy rather than traditional NSM with DIBR to explore the oncology safety of R-E-NSM with DIBR.
- Compared with traditional mastectomy, the medium-term oncology safety of R-E-NSM with DIBR was reliable.
- Compared with traditional mastectomy, the R-E-NSM with DIBR showed a lower overall complication, and there was no significant increase in the major complications.
- The surgical safety of R-E-NSM with DIBR is reliable, and patients can be safely discharged within 24 h, which can effectively save medical resources and not increase the psychological burden of patients.

For patients who cannot or do not want to undergo breast-conserving surgery (BCS), breast reconstruction can alleviate the psychological adverse effects of mastectomy^1,2^. However, traditional breast reconstruction surgery still leaves scars on the breast, which affects the cosmetic results of the breast^3^. Therefore, to reduce surgical trauma and improve the appearance of the reconstructed breast as much as possible, endoscopy-assisted or robot-assisted nipple-sparing mastectomy (E-NSM or R-NSM) with direct-to-implant breast reconstruction (DIBR) has attracted the attention of surgeons in recent decades^4,5^. However, traditional E-NSM or R-NSM with DIBR often requires multiple incisions and special instruments to overcome the difficulties of building working space and excising the gland, which increases the difficulty and trauma of surgery, prolongs the operation time and reduces the advantages of E-NSM or R-NSM with DIBR^5–8^. Therefore, the US Food and Drug Administration (FDA) has not included breast surgery as an indication for the da Vinci Surgical System^9^. This is also why E-NSM or R-NSM with DIBR is performed in only a small number of breast centres, let alone 24-h surgery centres.

However, in 2020, we innovatively conducted reverse-sequence endoscopic nipple-sparing mastectomy (R-E-NSM). The expansion tension of gas (CO_2_) and the use of the reverse dissection sequence method make the working space large and clear enough so that the dissociation of each plane becomes much more accessible (Supplemental Video 1, Supplemental Digital Content 8, http://links.lww.com/JS9/B834, Supplemental Digital Content 9, http://links.lww.com/JS9/B835), which significantly shortens the operation time without needing special instruments and effectively reduces the consumption of human resources (Supplemental Figure 1, Supplemental Digital Content 7, http://links.lww.com/JS9/B833)^10–15^. In addition to a lower rate of postoperative complications and the excellent aesthetic outcomes of reconstructed breasts, R-E-NSM is rapidly becoming popular in China^16,17^. We even conducted R-E-NSM with DIBR in the 24-h surgery centre and pioneered R-E-NSM and immediate breast reconstruction with reverse-sequence endoscopic latissimus dorsi muscle harvesting^12,18–20^.

In Supplemental Video 1, Supplemental Digital Content 8, http://links.lww.com/JS9/B834, Supplemental Digital Content 9, http://links.lww.com/JS9/B835, we can see that R-E-NSM is a procedure that is much the same as conventional NSM, except we adopt the reverse dissection sequence to conduct the surgery under the endoscope, so its oncologic safety is theoretically reliable. However, further data are needed to confirm this hypothesis. Therefore, the clinical data of patients with early breast cancer who underwent traditional open mastectomy or R-E-NSM with DIBR in our hospital from March 2020 to April 2022 were retrospectively analyzed to explore the medium-term oncological safety of R-E-NSM with DIBR. Moreover, to optimize hospital resource allocation^21,22^, we also examined the feasibility of discharging breast cancer patients who underwent R-E-NSM with DIBR within 24 h.

## Methods

### Ethical review

This study was approved by the Biomedical Ethics Committee of our Hospital (approval number: 2022-570) on 8 April 2022. The requirement for informed consent was waived, as this was a retrospective study. The study was registered in www.chictr.org.cn, which named a retrospective study comparing the safety outcomes of the reverse-sequence endoscopic nipple-sparing mastectomy and the conventional open breast surgery (RECBS-01). The work is in line with the STROCSS criteria^23^, Supplemental Digital Content 1, http://links.lww.com/JS9/B827. Informed consent forms were signed by the patients who allowed their procedures and postoperative results to be videoed and photographed.

### Study design and patients

In this single-centre retrospective cohort study, the clinical data of 3128 consecutive patients who underwent breast surgery at our hospital were retrospectively retrieved from a prospectively maintained database and screened for inclusion eligibility from 1 May 2020, to 30 April 2022^24^. After exclusion, according to the exclusion criteria, 1679 patients with breast cancer were considered eligible for this study. Patient clinical data, including demographic characteristics, preoperative imaging examinations, surgery, preoperative and postoperative treatments, and other related information, were collected.

Patients who were 18–70 years of age, no active smoking, had an American Society of Anesthesiologists status less than or equal to II, and no severe underlying disease (such as immunocompromised or poorly controlled diabetes mellitus, severe cardiopulmonary dysfunction, disorder of the coagulation mechanism or tendency to bleed, inability to tolerate general anaesthesia etc.) before surgery were included. Indications for R-E-NSM and traditional open mastectomy are the same in this study, including non-specific types of breast cancer, such as ductal carcinoma in situ, invasive ductal carcinoma, infiltrating lobular carcinoma; tumours less than or equal to 5 cm ( for patients with tumours > 5 cm, who can also be included if tumours ≤5 cm after neoadjuvant chemotherapy). Twenty-four-hour discharge patients should also meet the relevant requirements of our hospital and be accompanied by an adult at the time of discharge within 24 h after surgery. Exclusion criteria were breast cancer in pregnancy, tumours abutting the chest wall, skin (including inflammatory breast cancer), or nipple-areolar complex (NAC) (including Paget’s disease); grossly positive axillary involvement (N3); specific types of breast cancer, such as adenoid cystic carcinoma, malignant phyllodes tumours, sarcoma, etc.

In this study, patients were divided into the traditional open mastectomy (TOM) group and the R-E-NSM with DIBR (RE-R) group. The TOM group included only patients who underwent a modified radical mastectomy or total mastectomy with sentinel lymph node biopsy. BCS and simple NSM were also excluded from this study because the local recurrence rate in patients who undergo BCS is slightly higher than that in patients who undergo mastectomy^25–27^, and the incidence of postoperative complications of simple NSM is lower than that of NSM with DIBR.

Pathological staging was performed per the eighth edition of the American Joint Committee on Cancer (AJCC) TNM staging system for breast cancer. Preoperative and postoperative treatments were conducted per breast cancer treatment guidelines, including standard regimens and treatment courses.

### Propensity score matching (PSM)

The propensity score^28^, the conditional probability of being treated under the covariate condition, can reduce bias and equalize confounding factors between groups. In this study, we implemented PSM using the R package “MatchIt”16 version 4.1.2 with the following settings: 1:2 pairing, nearest neighbour methods, and a caliper of 0.02 to balance the baseline characteristics of the two groups.

### Follow-up

Postoperatively, patients not discharged within 24 h were regularly followed up at two weeks, one month, every 3–6 months for the first 5 years, and then annually thereafter. Patients who were discharged within 24 h were followed up via telephone call within the first 3 days after discharge and followed up in the outpatient clinic on the 4th day after discharge, afterward the same as patients who were not discharged within 24 h. The drainage tubes in patients in the TOM and RE-R groups were removed when drainage fluid was less than 20 ml/day or 50 ml/day for 3 consecutive days, respectively.

Recurrence and metastasis were defined according to whether either was based on computed tomography, magnetic resonance imaging, breast ultrasonography, pathological biopsy findings, and other data collected at the time of the patient’s visit. Death was defined as the receipt of a death certificate from the hospital, and the date of death was recorded based on the information provided by the patient’s family during the follow-up.

Local recurrence-free survival (LRFS) was defined as the time from the surgery date to recurrence in the ipsilateral chest wall or at the breast surgical site. Regional recurrence-free survival (RRFS) was defined as the time from the surgery date to recurrence in the ipsilateral axillary, internal mammary, or supraclavicular lymph node drainage area. Distant metastasis-free survival (DMFS) was defined as the time from surgery to recurrence at distant sites. Overall survival (OS) was defined as the time from the surgery date to death from any cause. Disease-free survival (DFS) was defined as the time from the surgery date to recurrence, metastasis, new breast cancer on the same or opposite side, and death from any cause. The primary study endpoints were LRFS, RRFS, DMFS, DFS, and OS.

The Clavien–Dindo classification (CDC) was developed to define and grade postoperative surgical complications^29^. Grades I or II were defined as minor complications, and grades III (those requiring surgical, endoscopic, or radiological intervention) or IV (those that are life-threatening) were defined as major complications. Postoperative lymphedema was classified as mild, moderate, and severe according to the degree of oedema.

Quality of life (QOL) is evaluated by BREAST-Q (version 2.0), which measures psychosocial well-being, sexual well-being, breast satisfaction, and chest physical well-being^30^. This survey uses Likert-scale responses ranging from 1 (not satisfied) to 5 (the most satisfied), which are converted to a standardized scale of 1–100. BREAST-Q scores between 1 month postoperatively and preoperatively were counted separately by questionnaire distribution. BREAST-Q outcome measures were calculated using the Q Score Scoring Software package.

### Statistical analysis

Categorical variables were compared using the χ^2^ test. Continuous variables were compared using a *t*-test. BREAST-Q scores were compared by the analysis of variance (ANOVA) F-test. Survival curves were estimated by the Kaplan–Meier method for LRFS, RRFS, DMFS, DFS, and OS, and the log-rank test was used for between-group comparisons. A two-tailed *P* value less than 0.05 was considered significant. R version 4.1.2 (R Foundation) and SPSS version 26 (IBM) were used for analyses. In this study, PSM was used to reduce bias and equalize confounding factors between groups.

## Results

**Figure 1:**
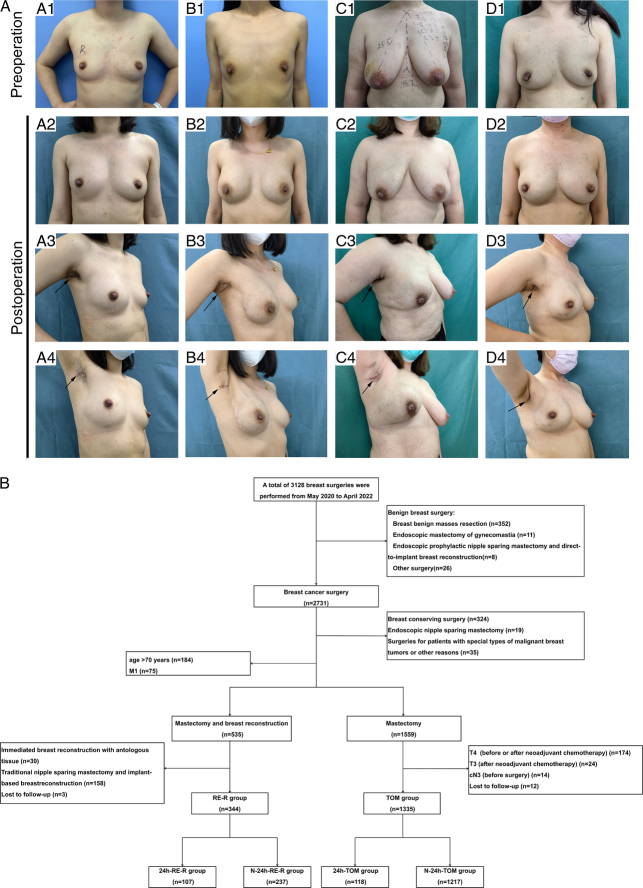
(A) Preoperative and postoperative images of the R-E-NSM with DIBR recipients. (A) (a1–4) Preoperative and 18-month postoperative images of a patient with right breast cancer who underwent subpectoral breast reconstruction. (b1–4) Preoperative and 12-month postoperative images of a patient with right breast cancer and BRCA2 gene mutation who underwent bilateral dual-plane breast reconstruction. (c1–4) Preoperative and 18-month postoperative images of a patient with right breast cancer who underwent prepectoral breast reconstruction. (d1–4) Preoperative and 18-month postoperative images of a patient with right breast cancer who underwent ipsilateral prepectoral breast reconstruction and contralateral breast augmentation. The arrows indicate the incisions. (B) The flowchart of data selection. 24 h-RE-R, RE-R patients discharged within 24 h; 24 h-TOM: traditional open mastectomy patients who were discharged within 24 h; N-24 h-RE-R, RE-R patients not discharged within 24 h; N-24 h-TOM: traditional open mastectomy patients who were not discharged within 24 h. RE-R, reverse-sequence endoscopic nipple-sparing mastectomy with direct-to-implant breast reconstruction; TOM: traditional open mastectomy.

A total of 1679 patients with breast cancer were considered eligible for this study. There were 1335 patients in the TOM group - 118 of them were discharged within 24 h (24 h-TOM), and 1217 patients were not discharged within 24 h (N-24 h-TOM). The RE-R group, on the other hand, had 344 patients - 107 patients were discharged within 24 h (24 h-RE-R), and 237 patients were not discharged within 24 h (N-24 h-RE-R). Figure 1A displays the preoperative and postoperative photographs of several patients who underwent R-E-NSM with DIBR. A flowchart of data selection is detailed in Figure 1B.

### Baseline characteristics of the TOM group and RE-R group before and after PSM

At baseline, the groups showed significant differences in age, BMI, hypertension status, surgery, axillary surgery, T stage, lymph node status, cancer stage, hormone receptor status, human epidermal growth factor receptor-2 (HER-2) status, adjuvant chemotherapy, adjuvant radiotherapy, and anti-HER-2 therapy. PSM was conducted between the two groups to reduce bias and equalize confounding factors. The propensity score covariates in this study included BMI, hypertension status, diabetes status, surgery, axillary surgery, T stage, lymph node status, cancer stage, hormone receptor status, HER-2 status, neoadjuvant chemotherapy, adjuvant endocrinotherapy, adjuvant chemotherapy, adjuvant radiotherapy, and anti-HER-2 therapy. After PSM, 887 patients were included in the study, including 572 patients in the TOM group and 315 in the RE-R group. Table 1 shows the baseline characteristics of the study population before and after PSM.

**Table 1:** Baseline characteristics of the traditional open mastectomy and reverse-sequence endoscopic nipple-sparing mastectomy with direct-to-implant breast reconstruction groups.

	Before PSM, *N* (%)			After PSM, *N* (%)		
Characteristic	TOM group (*n*=1335)	RE-R group (*n*=344)	*P*	TOM group (*n*=572)	RE-R group (*n*=315)	*P*
Age, year			<0.001			<0.001
<45	302 (22.6)	155 (45.1)		152 (26.6)	140 (44.4)	
≥45	1033 (77.4)	189 (54.9)		420 (73.4)	175 (55.6)	
BMI (kg/m^2^)			0.034			0.746
<24	870 (65.2)	245 (71.2)		399 (69.8)	223 (70.8)	
≥24	465 (34.8)	99 (28.8)		173 (30.2)	92 (29.2)	
Hypertension			<0.001			0.978
Yes	162 (12.1)	12 (3.5)		22 (3.8)	12 (3.8)	
No	1173 (87.9)	332 (96.5)		550 (96.2)	303 (96.2)	
Diabetes			0.036			0.184
Yes	55 (4.1)	6 (1.7)		5 (0.9)	6 (1.9)	
No	1280 (95.9)	338 (98.3)		567 (99.1)	309 (98.1)	
Surgery			0.175			0.574
Unilateral	1323 (99.1)	338 (98.3)		567 (99.1)	311 (98.7)	
Bilateral	12 (0.9)	6 (1.7)		5 (0.9)	4 (1.3)	
Axillary surgery			<0.001			0.293
SLNB	535 (40.1)	231 (67.2)		350 (61.2)	204 (64.8)	
ALND	800 (59.9)	113 (32.8)		222 (38.8)	111 (35.2)	
T stage (AJCC 8)			<0.001			0.858
Tis and T1	569 (42.6)	200 (58.1)		318 (55.6)	178 (56.5)	
T2	694 (52.0)	133 (38.7)		237 (41.4)	126 (40.0)	
T3	72 (5.4)	11 (3.2)		17 (3.0)	11 (3.5)	
Lymph node status			<0.001			0.349
Negative	836 (62.6)	263 (76.5)		410 (71.7)	235 (74.6)	
Positive	499 (37.4)	81 (23.5)		162 (28.3)	80 (25.4)	
Cancer stage (AJCC 8)			<0.001			0.876
0–I	454 (34.0)	171 (49.7)		273 (47.7)	149 (47.3)	
II	708 (53.0)	154 (44.8)		260 (45.5)	147 (46.7)	
III	173 (13.0)	19 (5.5)		39 (6.8)	19 (6.0)	
Hormone receptor status			0.002			0.608
Negative	379 (28.4)	66 (19.2)		129 (22.6)	62 (19.6)	
Positive	942 (70.6)	275 (79.9)		438 (76.5)	250 (79.4)	
Unknown	14 (1.0)	3 (0.3)		5 (0.9)	3 (1.0)	
HER-2 status			<0.001			0.983
Negative	915 (68.5)	272 (79.1)		445 (77.8)	246 (78.1)	
Positive	395 (29.6)	64 (18.6)		115 (20.1)	62 (19.7)	
Unknown	25 (1.9)	8 (2.3)		12 (2.1)	7 (2.2)	
Neoadjuvant chemotherapy			0.122			0.805
Yes	279 (20.9)	59 (17.2)		89 (15.6)	51 (16.2)	
No	1056 (79.1)	285 (82.8)		483 (84.4)	264 (83.8)	
Response evaluation of neoadjuvant chemotherapy^a^			0.075			0.188
CR+PR	256 (91.8)	58 (98.3)		80 (89.9)	50 (98.0)	
SD+PD	23 (8.2)	1 (1.7)		9 (10.1)	1 (2.0)	
Adjuvant chemotherapy			<0.001			0.182
Yes	1242 (93.0)	279 (81.1)		516 (90.2)	275 (87.3)	
No	93 (7.0)	65 (18.9)		56 (9.8)	40 (12.7)	
Adjuvant endocrinotherapy			0.005			0.454
Yes	926 (69.4)	265 (77.0)		434 (75.9)	246 (78.1)	
No	409 (30.6)	79 (23.0)		138 (24.1)	69 (21.9)	
Adjuvant radiotherapy			<0.001			0.898
Yes	483 (36.2)	85 (24.7)		153 (26.7)	83 (26.3)	
No	852 (63.8)	259 (75.3)		419 (73.3)	232 (73.7)	
Anti-HER-2 therapy			<0.001			0.768
Yes	372 (27.9)	59 (17.2)		99 (17.3)	57 (18.1)	
No	963 (72.1)	285 (82.8)		473 (82.7)	258 (81.9)	

ALND, axillary lymph node dissection; CR, complete response; HER-2, human epidermal growth factor receptor-2; PD, progressive disease; PR, partial response; PSM, propensity score matching; RE-R, reverse-sequence endoscopic nipple-sparing mastectomy with direct-to-implant breast reconstruction group; SD, stable disease; SLNB, sentinel lymph node biopsy; T stage, the tumour size before neoadjuvant chemotherapy and breast surgery; the positive of lymph node, the lymph node pathology is positive at any time; TOM, traditional open mastectomy group.

a Only for patients receiving neoadjuvant chemotherapy.

### Oncologic safety of the TOM group and RE-R group before and after PSM

**Figure 2:**
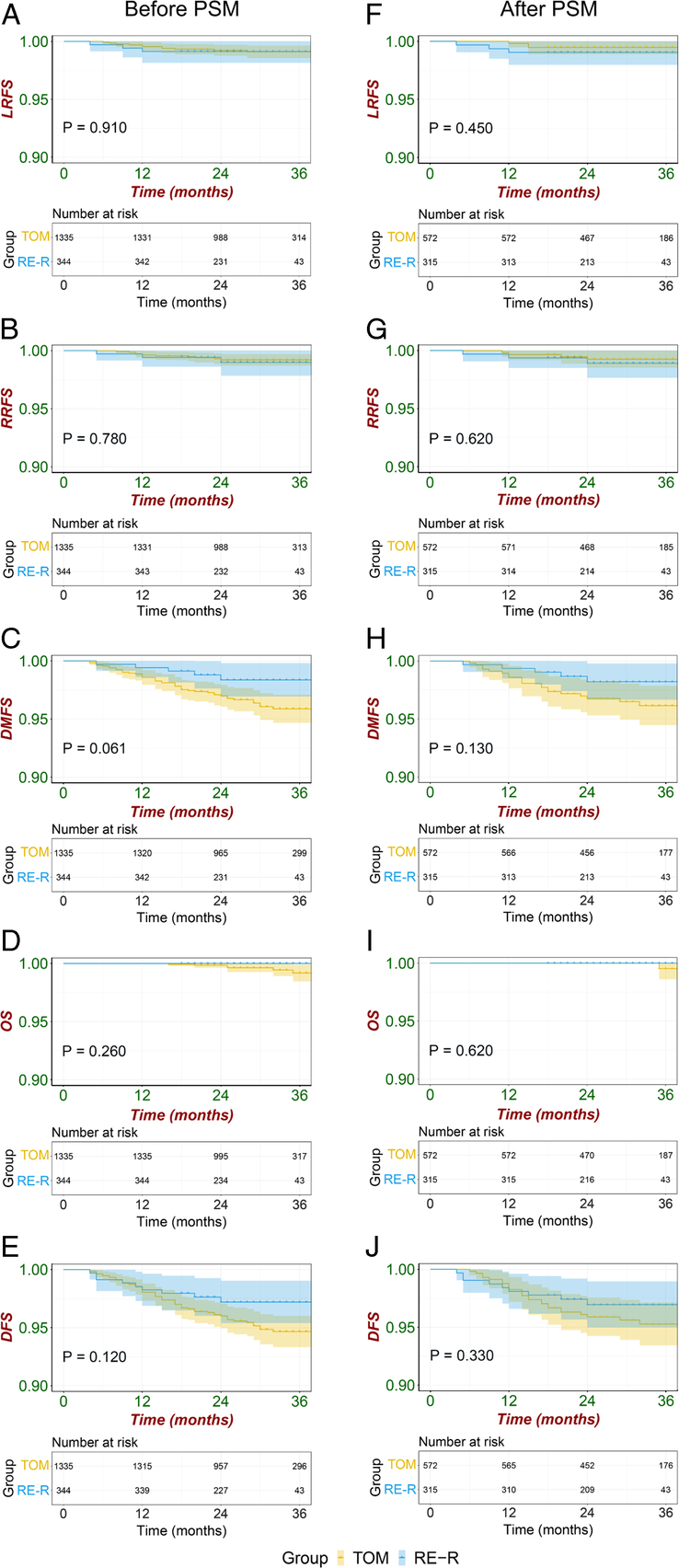
Kaplan–Meier survival curve before and after propensity score matching. (A,F) The LRFS between TOM group and RE-R group before and after PSM. (B,G) The RRFS between TOM group and RE-R group before and after PSM. (C,H) The DMFS between TOM group and RE-R group before and after PSM. (D,I) The OS between TOM group and RE-R group before and after PSM. (E,J) The DFS between TOM group and RE-R group before and after PSM. DFS, disease-free survival; DMFS, distant metastasis-free survival; LRFS, local recurrence-free survival; OS, overall survival; PSM, propensity score matching; RE-R, reverse-sequence endoscopic nipple-sparing mastectomy with direct-to-implant breast reconstruction; RRFS, regional recurrence-free survival; TOM, traditional open mastectomy.

The median [IQR] follow-up time was 30 [24–36] months [29 (23–33) months in the RE-R group and 30 (24–36) months in the TOM group]. Regarding before or after PSM, the *P* value of local recurrence-free survival (LRFS, 0.910 and 0.450), regional recurrence-free survival (RRFS, 0.780 and 0.620), distant metastasis-free survival (DMFS, 0.061 and 0.130), overall survival (OS, 0.260 and 0.620), disease-free survival (DFS, 0.120 and 0.330) were not significantly different between the RE-R group and the TOM group. The 3y-LRFS and 3y-DFS rates were 99.0% and 97.1% for the RE-R group and 99.5% and 95.3% for the TOM group, respectively. Figure 2 shows the medium-term survival results.

### Surgical safety of the TOM group and RE-R group

Before PSM, the RE-R group showed a significantly lower overall rate of complications (26.2% vs. 34.6%, *P* = 0.003) than the TOM group. However, there was no significant difference between the RE-R group (*n*=5, 1.5%) and the TOM group (*n*=15, 1.1%) in terms of the major complications rate (*P*=0.615). After PSM, the RE-R group still showed a lower overall complication rate than the TOM group (27.3% vs. 32.9%, *P* = 0.086), but there was no significant difference. Moreover, there was still no difference in the incidence of major complications between the two groups (1.6% vs. 0.7%, *P*=0.207), with an absolute difference of 0.9%. Table 2 shows the postoperative complications of the two groups.

**Table 2:** The postoperative complications of the traditional open mastectomy and reverse-sequence endoscopic nipple-sparing mastectomy with direct-to-implant breast reconstruction groups.

	Before PSM, *N* (%)			After PSM, *N* (%)		
Characteristic	TOM group (*n*=1335)	RE-R group (*n*=344)	*P*	TOM group (*n*=572)	RE-R group (*n*=315)	*P*
Postoperative complications^a^	462 (34.6)	90 (26.2)	0.003	188 (32.9)	86 (27.3)	0.086
Major complications^a^	15 (1.1)	5 (1.5)	0.615	4 (0.7)	5 (1.6)	0.207
Bleeding	2 (0.1)	0		1 (0.2)	0	
Wound disruption	3 (0.2)	2 (0.6)		1 (0.2)	2 (0.6)	
NAC ischaemia	0	0		0	0	
Flap ischaemia	7 (0.5)	0		2 (0.3)	0	
Infection	3 (0.2)	4 (1.2)		0	4 (1.3)	
Implant loss	—	3 (0.9)		0	3 (1.0)	
Minor complications^a^	257 (19.3)	37 (10.8)	<0.001	119 (20.8)	33 (10.5)	<0.001
seroma	235 (17.6)	22 (6.4)		107 (18.7)	20 (6.3)	
Bleeding	4 (0.3)	2 (0.6)		2 (0.3)	2 (0.6)	
Wound dehiscence	10 (0.7)	1 (0.3)		3 (0.5)	1 (0.3)	
NAC ischaemia	0	3 (0.9)		0	3 (1.0)	
Flap ischaemia	15 (1.1)	2 (0.6)		10 (1.7)	1 (0.3)	
Infection	8 (0.6)	9 (2.6)		5 (0.9)	8 (2.5)	
Lymphedema			0.710			0.415
Mild	184 (13.8)	48 (14.0)		65 (11.4)	47 (14.9)	
Moderate	44 (3.3)	8 (2.3)		15 (2.6)	8 (2.5)	
Sever	2 (0.1)	0		1 (0.2)	0	

NAC, nipple-areolar complex; PSM, propensity score matching; RE-R, reverse-sequence endoscopic nipple-sparing mastectomy with direct-to-implant breast reconstruction group; TOM, traditional open mastectomy group.

a Patients with ≥1 complication were computed once.

### Feasibility of 24-h discharge for RE-R patients

Similar to previous studies, we also found that the major or minor complications of 24 h-TOM and N-24 h-TOM were the same (Supplemental Table 1, Supplemental Digital Content 2, http://links.lww.com/JS9/B828 and 2, Supplemental Digital Content 3, http://links.lww.com/JS9/B829), suggesting that discharge of TOM patients within 24 h is safe in our institution. To further explore the feasibility of 24-h discharge for patients who have undergone R-E-NSM with DIBR, we compared the 24 h-RE-R group with the 24 h-TOM group and the N-24 h-RE-R group. Supplemental Table 3, Supplemental Digital Content 4, http://links.lww.com/JS9/B830 shows the baseline characteristics of the three groups.

The incidence rates of postoperative any complications in the 24 h-RE-R group, 24 h-TOM group, and N-24 h-RE-R group were 22.4%, 31.4%, and 27.8%, respectively. No significant differences existed between the 24 h-RE-R and 24 h-TOM groups (*P* =0.133) or the N-24 h-RE-R groups (*P* =0.290). Major complications occurred in 2 (1.9%) patients in the 24 h-RE-R group, 0 patients in the 24 h-TOM group, and 3 (1.3%) patients in the N-24 h-RE-R group. There was no significant difference between the 24 h-RE-R group and the N-24 h-RE-R group (*P*=0.665); only one patient (0.9%) suffered implant loss in the 24 h-RE-R group, and two (0.8%) suffered implant loss in the N-24 h-RE-R group. Notably, the absolute incidence of major complications in the 24 h-RE-R group was only 1.9% higher than that in the 24 h-TOM group, with no significant difference (*P*=0.136), and no emergency complications occurred in the RE-R group. Table 3 shows the incidence of postoperative complications in the three groups.

**Table 3:** The complications of the patients not discharged within 24 h after reverse-sequence endoscopic nipple-sparing mastectomy with direct-to-implant breast reconstruction group, patients discharged within 24 h after reverse-sequence endoscopic nipple-sparing mastectomy with direct-to-implant breast reconstruction group, and patients discharged within 24 h after traditional open mastectomy group.

Characteristic	24 h-RE-R group (*n*=107), *n* (%)	N-24 h-RE-R group (*n*=237), *n* (%)	24 h-TOM group (*n*=118), *n* (%)	*P*1 value	*P*2 value
Postoperative complications^a^	24 (22.4)	66 (27.8)	37 (31.4)	0.290	0.133
Major complications^a^	2 (1.9)	3 (1.3)	0	0.665	0.136
Bleeding	0	0	0		
Wound disruption	2 (1.9)	0	0		
NAC ischaemia	0	0	—		
Flap ischaemia	0	0	0		
Infection	1 (0.9)	3 (1.3)	0		
Implant loss	1 (0.9)	2 (0.8)	—		
Minor complications^a^	11 (10.3)	26 (11.0)	24 (20.3)	0.848	0.038
seroma	8 (7.5)	14 (5.9)	22 (18.6)		
Bleeding	1 (0.9)	1 (0.4)	1 (0.8)		
Wound disruption	0	1 (0.4	1 (0.8)		
NAC ischaemia	1 (0.9)	2 (0.8)	—		
Flap ischaemia	1 (0.9)	1 (0.4)	2 (1.7)		
Infection	1 (0.9)	8 (3.4)	2 (1.7)		
Lymphedema				0.398	0.459
Mild	13 (12.1)	35 (14.8)	14 (11.9)		
Moderate	1 (0.9)	7 (3.0)	4 (3.4)		
Sever	0	0	0		

The *P*1 value is the *P* of the N-24 h-RE-R group and 24 h-RE-R group. The *P*2 value is the *P* of the 24 h-RE-R group and 24 h-TOM group.

24 h-RE-R, patients discharged within 24 h after RE-R; 24 h-TOM, patients discharged within 24 h after; N-24 h-RE-R, patients not discharged within 24 h after RE-R; NAC, nipple-areolar complex; RE-R, reverse-sequence endoscopic nipple-sparing mastectomy with direct-to-implant breast reconstruction group; TOM, traditional open mastectomy.

a Patients with ≥1 complication were computed once.

In terms of quality of life, BREAST-Q questionnaires have been used to measure the psychological and life status of the patient. Regarding the RE-R group or TOM group, there were no significant differences between patients who were discharged within 24 h and those not discharged within 24 h in psychosocial well-being (*P*=0.576 and 0.420), sexual well-being (*P*=0.413 and 0.928), breast satisfaction (*P*=0.860 and 0.856) and chest well-being (*P*=0.424 and 0.655) aspects of the mean change between 1-month postoperative and preoperative breast-q score. Supplemental Table 4, Supplemental Digital Content 5, http://links.lww.com/JS9/B831 shows the BREAST-Q score of the two groups.

Regarding the RE-R group or TOM group, the length of hospital stay (both *P* values are less than 0.001 ) and hospitalization expenses (both *P* values are less than 0.001) in patients discharged within 24 h are significantly lower than those not discharged within 24 h. Supplemental Table 5, Supplemental Digital Content 6, http://links.lww.com/JS9/B832 shows the length of hospital stay and hospitalization expenses of the two groups.

## Discussion

R-E-NSM addresses the limitations of previous E-NSM and R-NSM (not only optimizing the procedure so that it does not require the use of any special equipment but also shortening the operation time, reducing emergent complications and early surgical complications, and broadening the indications for endoscopic breast reconstruction. Therefore, R-E-NSM with DIBR has become increasingly popular in China in the recent three years^10–12,15,18,19^. In this study, to explore the oncological safety of R-E-NSM with DIBR, we compared it with traditional open mastectomy after excluding BCS due to its slightly high local recurrence rate. After PSM, LRFS, RRFS, DMFS, OS, and DFS were not significantly different between the RE-R and TOM groups over a median follow-up time of more than 2 years. Moreover, we compared the 24 h-RE-R group with the N-24 h-RE-R group and the 24 h-TOM group and found no significant differences in the overall incidence of surgical complications or major complications among them. Most importantly, the absolute incidence of major complications in the 24 h-RE-R group was only 1.9% higher than that in the 24 h-TOM group, with no significant difference and no complications requiring emergency intervention. Thus, we believe that the medium-term oncological safety of R-E-NSM with DIBR is high and that patients who have undergone R-E-NSM with DIBR can be safely discharged within 24 h.

At present, liposuction has been widely used in endoscopy-assisted breast reconstruction surgery^17,31^, but some scholars still doubt its oncology safety^32,33^. In addition, some scholars have proposed that transaxillary endoscopy-assisted NSM will result in incomplete resection of the gland in the lower-inner quadrant of the breast, thus also increasing the risk of local recurrence^34^. In our study, we abandoned the liposuction method and adopted the nonliposuction approach to build the working space. The reverse-sequence method improves the visibility of the area under the endoscope, and the CO_2_ pressure, just like a universal retractor, makes us adopt an electric scalpel to dissect all the planes, which is the same as conventional NSM^10–12,18,19^, thus theoretically dispelling concerns about the oncological safety of liposuction. Meanwhile, when dissociating the superficial layer of superficial fascia in the lower-inner quadrant of the breast, the surgeon makes a small incision of ~2 mm, known as “HUAXI Hole 1”, at the edge of the upper-outer quadrant of the areola, which has the same advantages as the double incisions but without any disadvantages^35–38^. The breast glands are entirely removed, regardless of whether the tumour is in the lower-inner quadrant. Therefore, the excision area of the RE-R group is consistent with that of the TOM group, and its local oncology safety is reliable.

Since the patients in the RE group were significantly younger than those in the TOM group, we did not perform PSM matching for the age of the patients in order to prevent a large number of patient loss. Previous studies have shown that young breast cancer is more aggressive and has a higher recurrence rate^39,40^. This study showed no significant difference in LRFS between the two groups, though the mean age of the RE-R group is younger than that of the TOM group. Therefore, we believe that the medium-term local oncology safety of the RE group is relatively reliable.

In this study, we also found no significant difference in RRFS between the TOM and RE-R groups due to direct vision for axillary surgery. In the comparison, DFS and MDFS were found to be higher in the RE-R group than in the TOM group, but the difference was not significant. This may be due to reduced tumour compression during endoscopic surgery, which may reduce the risk of tumour metastasis by reducing circulating tumour cells, but further research is needed^31^. There was no significant difference in OS between the two groups, possibly due to the same treatment effect or the possibility that the follow-up time was too short in the RE-R and TOM groups.

To explore the surgical safety of R-E-NSM with DIBR, we also compared it with traditional open mastectomy. We found that the incidence of postoperative complications was lower in the RE-R group than that in the TOM group. Because the flap of patients with mastectomy wounds is large and must be attached to the chest wall after the operation. Therefore, any seroma that causes the flap to float needs to be managed in patients who undergo mastectomy. However, in R-E-NSM with DIBR, postoperative seroma does not require management if there are no signs of infection (such as red skin) or compression symptoms. Studies have shown that implant-based breast reconstruction has a higher incidence of infection than traditional mastectomy due to the implant^41,42^. However, the RE-R group’s infection rate is very low because the R-E-NSM procedure is considered a “no touch” procedure with a short operation time. Meanwhile, R-E-NSM procedure also minimizes incisions on the breast (only a 2 mm Huaxi hole 1), eliminating the impact on the blood supply of the flap and the NAC and reducing the risk of necrosis of the NAC or skin flap.

Many studies have confirmed that it is safe for breast cancer patients to be discharged within 24 h after mastectomy^21,43–46^, which were the same as our study. However, it is less feasible to discharge patients who have undergone implant-based breast reconstruction within 24 h at breast centres worldwide, let alone E-NSM with DIBR. In this study, when comparing the 24 h-RE-R group with the N-24 h-RE-R group as well as with the 24 h-TOM group, we found no significant differences in the incidences of postoperative complications, and the absolute incidence of major complications was only 1.9% for patients who were discharged within 24 h after RE-R. There were also no complications that needed emergency intervention. Thus, as a highly efficient surgery, RE-R allows the safe discharge of patients within 24 h. At the same time, we also found that discharging within 24 h did not affect the patient’s quality of life. Still, it significantly shortened the length of stay and saved hospitalization expenses like other studies^47,48^.

This study is the first to explore the median oncologic and surgical safety of our novel R-E-NSM with DIBR procedure. However, this was a single-centre retrospective study. Although the propensity score matching method was used to reduce bias between the two groups, the possibility of selection bias could not be entirely eliminated. In addition, the median follow-up time in this study was 30 months, which is slightly short and needs further extension to demonstrate the oncological safety of R-E-NSM with DIBR. In the future, multicenter prospective large-sample studies with longer follow-up periods will be needed to confirm the long-term oncological safety of R-E-NSM with DIBR.

## Conclusion

R-E-NSM with DIBR is an innovative oncologic surgical procedure that not only improves cosmetic outcomes but also ensures reliable oncologic safety and fewer complications, enabling patients to be safely discharged within 24 h. A long-term prospective multicenter assessment will be supporting.

## Ethical approval

This study was approved by the Biomedical Ethics Committee of West China Hospital, Sichuan University (approval number: 2022-570) on 8 April 2022.

## Consent

This study was approved by the Biomedical Ethics Committee of West China Hospital, Sichuan University (approval number: 2022-570) on 8 April 2022. The requirement for informed consent was waived, as this was a retrospective study. Informed consent forms were signed by the patients who allowed their procedures and postoperative results to be videoed and photographed. A copy of the written consent is available for review by the Editor-in-Chief of this journal on request.

## Sources of funding

This work was supported by the Key Projects of the Sichuan Provincial Health Commission (21PJ042), Incubation Project of West China Hospital of Sichuan University (2022HXFH004); Natural Science Foundation of Sichuan Province (22NSFSC2361).

## Author contribution

J.Z.: investigation, formal analysis, writing—original draft. Y.X.: investigation, formal analysis. F.L.: formal analysis, writing—original draft. Y.F.: investigation. H.Y.: investigation. M.Q.: investigation. Q.Z.: investigation. K.C.: investigation. H.D.: investigation. Y.L.: methodology, investigation. P.L.: methodology, investigation. Z.D.: conceptualization, supervision, writing—review and editing.

## Conflicts of interest disclosure

The authors declare that they have no potential conflicts of interest to disclose.

## Research registration unique identifying number (UIN)

1.Name of the registry: Chinese clinical trial registry.
2.Unique Identifying number or registration ID: ChiCTR2300075286.
Hyperlink to your specific registration (must be publicly accessible and will be checked): https://www.chictr.org.cn/bin/project/edit?pid=202541.

## Guarantor

Zhenggui Du.

## Data availability statement

Datasets generated during and/or analyzed during the current study are not publicly available, but available upon reasonable request.

## Provenance and peer review

Not commissioned, externally peer-reviewed.

## Supplementary Material

SUPPLEMENTARY MATERIAL
