# Supplementary material for: A novel technique of reverse-sequence endoscopic nipple-sparing mastectomy with direct-to-implant breast reconstruction: medium-term oncological safety outcomes and feasibility of 24-h discharge for breast cancer patients
Source: Int J Surg. 2024 Feb 9;110(4):2243–52. doi: 10.1097/JS9.0000000000001134 (PMC11020081; doi:10.1097/JS9.0000000000001134)
Supplement: SUPPLEMENTARY MATERIAL [file js9-110-2243-s002.docx]

Paper title: A novel technique of reverse-sequence endoscopic nipple-sparing mastectomy with direct-to-implant breast reconstruction: medium-term oncological safety outcomes and feasibility of 24-hour discharge for breast cancer patients

First author: Jiao Zhou

Supplemental Table 1. Baseline characteristics of the traditional open mastectomy patients

| Characteristic | N-24 h-TOM group  (n=1217) | 24 h-TOM group  (n=118) | P value |
| --- | --- | --- | --- |
| Age, y |  |  | 0.446 |
| <45 | 272(22.4%) | 30(25.4%) |  |
| ≥45 | 945(77.6%) | 88(74.6%) |  |
| BMI (kg/m^2^) |  |  | 0.217 |
| <24 | 787(64.7%) | 83(70.3%) |  |
| ≥24 | 430(35.3%) | 35(29.7%) |  |
| Hypertension |  |  | 0.327 |
| Yes | 151(12.4%) | 11(9.3%) |  |
| No | 1066(87.6%) | 107(90.7%) |  |
| Diabetes |  |  | 0.128 |
| Yes | 47(3.9%) | 8(6.8%) |  |
| No | 1170(96.1%) | 110(93.2%) |  |
| Surgery |  |  | 0.279 |
| Unilateral | 1205(99.0%) | 118(100%) |  |
| Bilateral | 12(1.0%) | 0 |  |
| Axillary surgery |  |  | <0.001 |
| SLNB | 469(38.5%) | 66(55.9%) |  |
| ALND | 748(61.5%) | 52(44.1%) |  |
| T stage (p AJCC 8) |  |  | 0.112 |
| Tis and T1 | 508(41.7%) | 61(51.7%) |  |
| T2 | 642(52.8%) | 52(44.1%) |  |
| T3 | 67(5.5%) | 5(4.2%) |  |
| Lymph node status |  |  | 0.309 |
| Negative | 757(62.2%) | 79(66.9%) |  |
| Positive | 460(37.8%) | 39(33.1%) |  |
| Cancer stage (AJCC 8) |  |  | 0.462 |
| 0-I | 408(33.5%) | 46(39.0%) |  |
| II | 649(53.3%) | 59(50.0%) |  |
| III | 160(13.2%) | 13(11.0%) |  |
| Hormone receptor status |  |  | 0.002 |
| Negative | 346(28.4%) | 33(28.0%) |  |
| Positive | 862(70.9%) | 80(67.8%) |  |
| Unknown | 9(0.7%) | 5(4.2%) |  |
| HER-2 status |  |  | <0.001 |
| Negative | 833(68.4%) | 82(69.5%) |  |
| Positive | 367(30.2%) | 28(23.7%) |  |
| Unknown | 17(1.4%) | 8(6.8%) |  |
| Neoadjuvant chemotherapy |  |  | 0.179 |
| Yes | 260(21.4%) | 19(16.1%) |  |
| No | 957(78.6%) | 99(83.9%) |  |
| Response evaluation of neoadjuvant chemotherapy* |  |  | 0.708 |
| CR+PR | 239(91.9%) | 17(89.5%) |  |
| SD+PD | 21(8.1%) | 2(10.5%) |  |
| Adjuvant chemotherapy |  |  | 0.500 |
| Yes | 1134(93.2%) | 108(91.5%) |  |
| No | 83(6.8%) | 10(8.5%) |  |
| Adjuvant endocrinotherapy |  |  | 0.421 |
| Yes | 848(69.7%) | 78(66.1%) |  |
| No | 369(30.3%) | 40(33.9%) |  |
| Adjuvant radiotherapy |  |  | 0.346 |
| Yes | 445(36.6%) | 38(32.2%) |  |
| No | 772(63.4%) | 80(67.8%) |  |
| Anti-HER-2 therapy |  |  | 0.056 |
| Yes | 348(28.6%) | 24(20.3%) |  |
| No | 869(71.4%) | 94(79.7%) |  |

*Only for patients receiving neoadjuvant chemotherapy. TOM: traditional open mastectomy, 24 h-TOM: patients discharged within 24 hours after TOM, N-24 h-TOM: patients not discharged within 24 hours after TOM, BMI: body mass index, SLNB: sentinel lymph node biopsy, ALND: axillary lymph node dissection, CR: complete response, PR: partial response, SD: stable disease, PD: progressive disease, HER-2: human epidermal growth factor receptor-2, T stage: the tumor size before neoadjuvant chemotherapy and breast surgery, the positive of lymph node: the lymph node pathology is positive at any time.
