# Supplementary material for: A novel technique of reverse-sequence endoscopic nipple-sparing mastectomy with direct-to-implant breast reconstruction: medium-term oncological safety outcomes and feasibility of 24-h discharge for breast cancer patients
Source: Int J Surg. 2024 Feb 9;110(4):2243–52. doi: 10.1097/JS9.0000000000001134 (PMC11020081; doi:10.1097/JS9.0000000000001134)
Supplement: SUPPLEMENTARY MATERIAL [file js9-110-2243-s003.docx]

Paper title: A novel technique of reverse-sequence endoscopic nipple-sparing mastectomy with direct-to-implant breast reconstruction: medium-term oncological safety outcomes and feasibility of 24-hour discharge for breast cancer patients

First author: Jiao Zhou

Supplemental Table 2. Postoperative complications of the traditional open mastectomy group patients.

| Characteristic | N-24 h-TOM group  (n=1217) | 24 h-TOM group  (n=118) | P value |
| --- | --- | --- | --- |
| Postoperative complications* | 425(34.9%) | 37(31.4%) | 0.437 |
| Major complications* | 15(1.2%) | 0 | 0.225 |
| Bleeding | 2(0.2%) | 0 |  |
| Wound disruption | 3(0.2%) | 0 |  |
| Flap ischemia | 7(0.6%) | 0 |  |
| Infection | 3(0.2%) | 0 |  |
| Minor complications* | 233(19.1%) | 24(20.3%) | 0.754 |
| Seroma | 213(17.5%) | 22(18.6%) |  |
| Bleeding | 3(0.2%) | 1(0.8%) |  |
| Wound dehiscence | 9(0.7%) | 1(0.8%) |  |
| Flap ischemia | 13(1.1%) | 2(1.7%) |  |
| Infection | 6(0.5%) | 2(1.7%) |  |
| Lymphedema |  |  | 0.896 |
| Mild | 170(14.0%) | 14(11.9%) |  |
| Moderate | 40(3.3%) | 4(3.4%) |  |
| Sever | 2(0.2%) | 0 |  |

* Patients with ≥1 complication were counted once. TOM: traditional open mastectomy, 24 h-TOM: patients discharged within 24 hours after TOM, N-24 h-TOM: patients not discharged within 24 hours after TOM.
