# Supplementary material for: A novel technique of reverse-sequence endoscopic nipple-sparing mastectomy with direct-to-implant breast reconstruction: medium-term oncological safety outcomes and feasibility of 24-h discharge for breast cancer patients
Source: Int J Surg. 2024 Feb 9;110(4):2243–52. doi: 10.1097/JS9.0000000000001134 (PMC11020081; doi:10.1097/JS9.0000000000001134)
Supplement: SUPPLEMENTARY MATERIAL [file js9-110-2243-s004.docx]

Paper title: A novel technique of reverse-sequence endoscopic nipple-sparing mastectomy with direct-to-implant breast reconstruction: medium-term oncological safety outcomes and feasibility of 24-hour discharge for breast cancer patients

First author: Jiao Zhou

Supplemental Table 3. Baseline characteristics of the patients discharged within 24 hours after reverse-sequence endoscopic nipple-sparing mastectomy with direct-to-implant breast reconstruction group, patients not discharged within 24 hours after reverse-sequence endoscopic nipple-sparing mastectomy with direct-to-implant breast reconstruction group and patients discharged within 24 hours after traditional open mastectomy group

| Characteristic | 24 h-RE-R group (n=107) | N-24 h-RE-R group (n=237) | 24 h-TOM group (n=118) | P1 value | P2 value |
| --- | --- | --- | --- | --- | --- |
| Age, y |  |  |  | 0.375 | <0.001 |
| <45 | 52(48.6%) | 103(43.5%) | 30(25.4%) |  |  |
| ≥45 | 55(51.4%) | 134(56.5%) | 88(74.6%) |  |  |
| BMI (kg/m^2^) |  |  |  | 0.180 | 0.521 |
| <24 | 71(66.4%) | 174(73.4%) | 83(70.3%) |  |  |
| ≥24 | 36(33.6%) | 63(26.6%) | 35(29.7%) |  |  |
| Hypertension |  |  |  | 0.150 | 0.292 |
| Yes | 6(5.6%) | 6(2.5%) | 11(9.3%) |  |  |
| No | 101(94.4%) | 231(97.5%) | 107(90.7%) |  |  |
| Diabetes |  |  |  | 0.058 | 0.311 |
| Yes | 4(3.7%) | 2(0.8%) | 8(6.8%) |  |  |
| No | 103(96.3%) | 235(99.2%) | 110(93.2%) |  |  |
| Surgery |  |  |  | 0.097 | 1.000 |
| Unilateral | 107(100%) | 231(97.5%) | 118(100%) |  |  |
| Bilateral | 0 | 6(2.5%) | 0 |  |  |
| Axillary surgery |  |  |  | 0.435 | 0.028 |
| SLNB | 75(70.1%) | 156(65.8%) | 66(55.9%) |  |  |
| ALND | 32(29.9%) | 81(34.2%) | 52(44.1%) |  |  |
| T stage (p AJCC 8) |  |  |  | 0.621 | 0.447 |
| Tis and T1 | 62(57.9%) | 138(58.2%) | 61(51.7%) |  |  |
| T2 | 43(40.2%) | 90(38.0%) | 52(44.1%) |  |  |
| T3 | 2(1.9%) | 9(3.8%) | 5(4.2%) |  |  |
| Lymph node status |  |  |  | 0.957 | 0.108 |
| Negative | 82(76.6%) | 181(76.4%) | 79(66.9%) |  |  |
| Positive | 25(23.4%) | 56(23.6%) | 39(33.1%) |  |  |
| Cancer stage (AJCC 8) |  |  |  | 0.206 | 0.173 |
| 0-I | 55(51.4%) | 116(48.9%) | 46(39.0%) |  |  |
| II | 43(40.2%) | 111(46.8%) | 59(50.0%) |  |  |
| III | 9(8.4%) | 10(4.2%) | 13(11.0%) |  |  |
| Hormone receptor status |  |  |  | 0.323 | 0.065 |
| Negative | 18(16.8%) | 48(20.3%) | 33(28.0%) |  |  |
| Positive | 87(81.3%) | 188(79.3%) | 80(67.8%) |  |  |
| Unknown | 2(1.9%) | 1(0.4%) | 5(4.2%) |  |  |
| HER-2 status |  |  |  | 0.502 | 0.343 |
| Negative | 83(77.6%) | 189(79.7%) | 82(69.5%) |  |  |
| Positive | 20(18.7%) | 44(18.6%) | 28(23.7%) |  |  |
| Unknown | 4(3.7%) | 4(1.7%) | 8(6.8%) |  |  |
| Neoadjuvant chemotherapy |  |  |  | 0.098 | 0.397 |
| Yes | 13(12.1%) | 46(19.4%) | 19(16.1%) |  |  |
| No | 94(87.9%) | 191(80.6%) | 99(83.9%) |  |  |
| Response evaluation of neoadjuvant chemotherapy* |  |  |  | 0.592 | 0.227 |
| CR+PR | 13(100%) | 45(97.8%) | 17(89.5%) |  |  |
| SD+PD | 0 | 1(2.2%) | 2(10.5%) |  |  |
| Adjuvant chemotherapy |  |  |  | 0.509 | 0.058 |
| Yes | 89(83.2%) | 190(80.2%) | 108(91.5%) |  |  |
| No | 18(16.8%) | 47(19.8%) | 10(8.5%) |  |  |
| Adjuvant endocrinotherapy |  |  |  | 0.123 | 0.006 |
| Yes | 88(82.2%) | 177(74.7%) | 78(66.1%) |  |  |
| No | 19(17.8%) | 60(25.3%) | 40(33.9%) |  |  |
| Adjuvant radiotherapy |  |  |  | 0.698 | 0.140 |
| Yes | 25(23.4%) | 60(25.3%) | 38(32.2%) |  |  |
| No | 82(76.6%) | 177(74.7%) | 80(67.8%) |  |  |
| Anti-HER-2 therapy |  |  |  | 0.300 | 0.211 |
| Yes | 15(14.0%) | 44(18.6%) | 24(20.3%) |  |  |
| No | 92(86.0%) | 193(81.4%) | 94(79.7%) |  |  |

*Only for patients receiving neoadjuvant chemotherapy. TOM: traditional open mastectomy, 24 h-TOM: patients discharged within 24 hours after TOM, RE-R: reverse-sequence endoscopic nipple-sparing mastectomy with direct-to-implant breast reconstruction group, 24 h-RE-R: patients discharged within 24 hours after RE-R, N-24 h-RE-R: patients not discharged within 24 hours after RE-R, BMI: body mass index, SLNB: sentinel lymph node biopsy, ALND: axillary lymph node dissection, CR: complete response, PR: partial response, SD: stable disease, PD: progressive disease, HER-2: human epidermal growth factor receptor-2. The P1 value is the P of the N-24 h-RE-R group and 24 h-RE-R group. The P2 value is the P of the 24 h-RE-R group and 24 h-TOM group, T stage: the tumor size before neoadjuvant chemotherapy and breast surgery, the positive of lymph node: the lymph node pathology is positive at any time.
