# Supplementary material for: A novel technique of reverse-sequence endoscopic nipple-sparing mastectomy with direct-to-implant breast reconstruction: medium-term oncological safety outcomes and feasibility of 24-h discharge for breast cancer patients
Source: Int J Surg. 2024 Feb 9;110(4):2243–52. doi: 10.1097/JS9.0000000000001134 (PMC11020081; doi:10.1097/JS9.0000000000001134)
Supplement: SUPPLEMENTARY MATERIAL [file js9-110-2243-s005.docx]

Paper title: A novel technique of reverse-sequence endoscopic nipple-sparing mastectomy with direct-to-implant breast reconstruction: medium-term oncological safety outcomes and feasibility of 24-hour discharge for breast cancer patients

First author: Jiao Zhou

|  | 24 h-TOM group | N-24 h-TOM group | P1 value | 24 h-RE-R group | N-24 h-RE-R group | P2 value |
| --- | --- | --- | --- | --- | --- | --- |
| Psychosocial well-being | -18.87±15.373 | -17.69±12.913 | 0.420 | -11.25±9.610 | -12.00±12.046 | 0.576 |
| Sexual well-being | -20.47±15.052 | -20.63±17.613 | 0.928 | -10.03±17.907 | -11.66±16.320 | 0.413 |
| Breast satisfaction | -22.49±12.893 | -22.26±16.243 | 0.856 | -8.701±9.877 | -8.50±9.408 | 0.860 |
| Chest well-being | -13.66±16.770 | -12.96±16.217 | 0.655 | -11.27±12.917 | -12.51±13.149 | 0.424 |

Supplemental Table 4. Mean change between 1-month postoperative and preoperative breast-q score of the traditional open mastectomy and reverse-sequence endoscopic nipple-sparing mastectomy with direct-to-implant breast reconstruction groups.

TOM: traditional open mastectomy, 24 h-TOM: patients discharged within 24 hours after TOM, N-24 h-TOM: patients not discharged within 24 hours after TOM, RE-R: reverse-sequence endoscopic nipple-sparing mastectomy with direct-to-implant breast reconstruction group, 24 h-RE-R: patients discharged within 24 hours after RE-R, N-24 h-RE-R: patients not discharged within 24 hours after RE-R, P1: the P value of 24 h-TOM and N-24 h-TOM groups, P2: the P value of 24 h-RE-R and N-24 h-RE-R groups.
