# Supplementary material for: A novel technique of reverse-sequence endoscopic nipple-sparing mastectomy with direct-to-implant breast reconstruction: medium-term oncological safety outcomes and feasibility of 24-h discharge for breast cancer patients
Source: Int J Surg. 2024 Feb 9;110(4):2243–52. doi: 10.1097/JS9.0000000000001134 (PMC11020081; doi:10.1097/JS9.0000000000001134)
Supplement: SUPPLEMENTARY MATERIAL [file js9-110-2243-s006.docx]

Paper title: A novel technique of reverse-sequence endoscopic nipple-sparing mastectomy with direct-to-implant breast reconstruction: medium-term oncological safety outcomes and feasibility of 24-hour discharge for breast cancer patients

First author: Jiao Zhou

|  | 24 h-TOM group | N-24 h-TOM group | P1 value | 24 h-RE-R group | N-24 h-RE-R group | P2 value |
| --- | --- | --- | --- | --- | --- | --- |
| Length of stay (day) | 1.00±0.000 | 6.30±0.889 | < 0.001 | 1.00±0.000 | 6.39±0.898 | < 0.001 |
| Hospitalization expenses (RMB) | 10971.94±1903.973 | 16235.62±4125.838 | < 0.001 | 50815.33±3909.712 | 55351.68±7156.681 | < 0.001 |

Supplemental Table 5. Length of stay and hospitalization expenses of the traditional open mastectomy and reverse-sequence endoscopic nipple-sparing mastectomy with direct-to-implant breast reconstruction groups.

TOM: traditional open mastectomy, 24 h-TOM: patients discharged within 24 hours after TOM, N-24 h-TOM: patients not discharged within 24 hours after TOM, RE-R: reverse-sequence endoscopic nipple-sparing mastectomy with direct-to-implant breast reconstruction group, 24 h-RE-R: patients discharged within 24 hours after RE-R, N-24 h-RE-R: patients not discharged within 24 hours after RE-R, P1: the P value of 24 h-TOM and N-24 h-TOM groups, P2: the P value of 24 h-RE-R and N-24 h-RE-R groups.
