# Supplementary material for: A novel technique of reverse-sequence endoscopic nipple-sparing mastectomy with direct-to-implant breast reconstruction: medium-term oncological safety outcomes and feasibility of 24-h discharge for breast cancer patients
Source: Int J Surg. 2024 Feb 9;110(4):2243–52. doi: 10.1097/JS9.0000000000001134 (PMC11020081; doi:10.1097/JS9.0000000000001134)

Paper title: A novel technique of reverse-sequence endoscopic nipple-sparing mastectomy with direct-to-implant breast reconstruction: medium-term oncological safety outcomes and feasibility of 24-hour discharge for breast cancer patients

First author: Jiao Zhou

Supplemental Figure 1


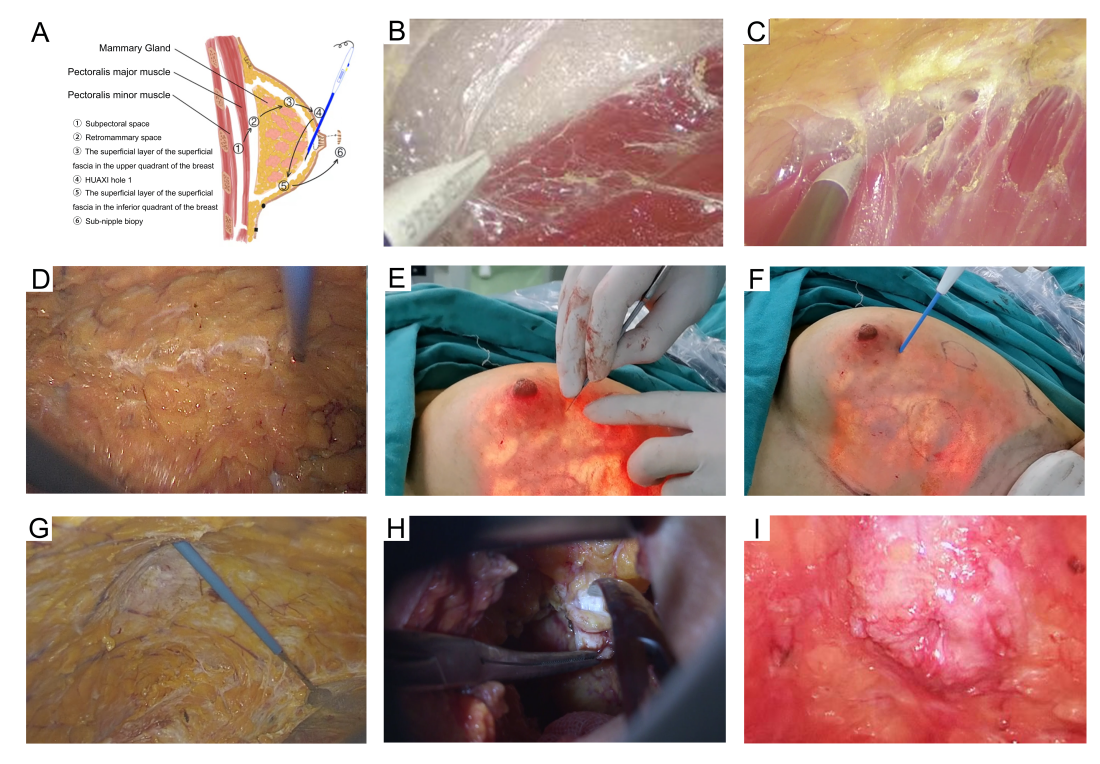

Supplement: SUPPLEMENTARY MATERIAL [file js9-110-2243-s007.docx]
