# Supplementary material for: A novel technique of reverse-sequence endoscopic nipple-sparing mastectomy with direct-to-implant breast reconstruction: medium-term oncological safety outcomes and feasibility of 24-h discharge for breast cancer patients
Source: Int J Surg. 2024 Feb 9;110(4):2243–52. doi: 10.1097/JS9.0000000000001134 (PMC11020081; doi:10.1097/JS9.0000000000001134)
Supplement: SUPPLEMENTARY MATERIAL [file js9-110-2243-s009.docx]

**Supplemental video 1:**

Name: Step of reverse-sequence endoscopic nipple-sparing mastectomy with direct-to-implant breast reconstruction

Author: Jiao Zhou

Photographer: Jiao Zhou

Participant: Zhenggui Du and patients

Length: 4min

Size: 96.4MB
